# Host-Associated and Free-Living Phage Communities Differ Profoundly in Phylogenetic Composition

**DOI:** 10.1371/journal.pone.0016900

**Published:** 2011-02-24

**Authors:** J. Gregory Caporaso, Rob Knight, Scott T. Kelley

**Affiliations:** 1 Department of Chemistry and Biochemistry, University of Colorado at Boulder, Boulder, Colorado, United States of America; 2 Howard Hughes Medical Institute, Boulder, Colorado, United States of America; 3 Department of Biology, San Diego State University, San Diego, California, United States of America; Argonne National Laboratory, United States of America

## Abstract

Phylogenetic profiling has been widely used for comparing bacterial communities, but has so far been impossible to apply to viruses because of the lack of a single marker gene analogous to 16S rRNA. Here we developed a reference tree approach for matching viral sequences and applied it to the largest viral datasets available. The resulting technique, Shotgun UniFrac, was used to compare host-associated and non-host-associated phage communities (130 total metagenomes), and revealed a profound split similar to that found with bacterial communities. This new informatics approach complements analysis of bacterial communities and promises to provide new insights into viral community dynamics, such as top-down versus bottom-up control of bacterial communities by viruses in a range of systems.

## Introduction

The phylogenetic composition of bacterial communities is primarily determined by whether they are found in host-associated or free-living environments [1]. Much less is known about the phylogenetic composition of viral communities, which may comprise most of the genetic diversity on Earth. If viral communities follow this pattern, microbial and viral community composition should be correlated, adding to recent evidence that phage predation can exert top-down control on microbial communities [2], [3].

**Figure 1:**
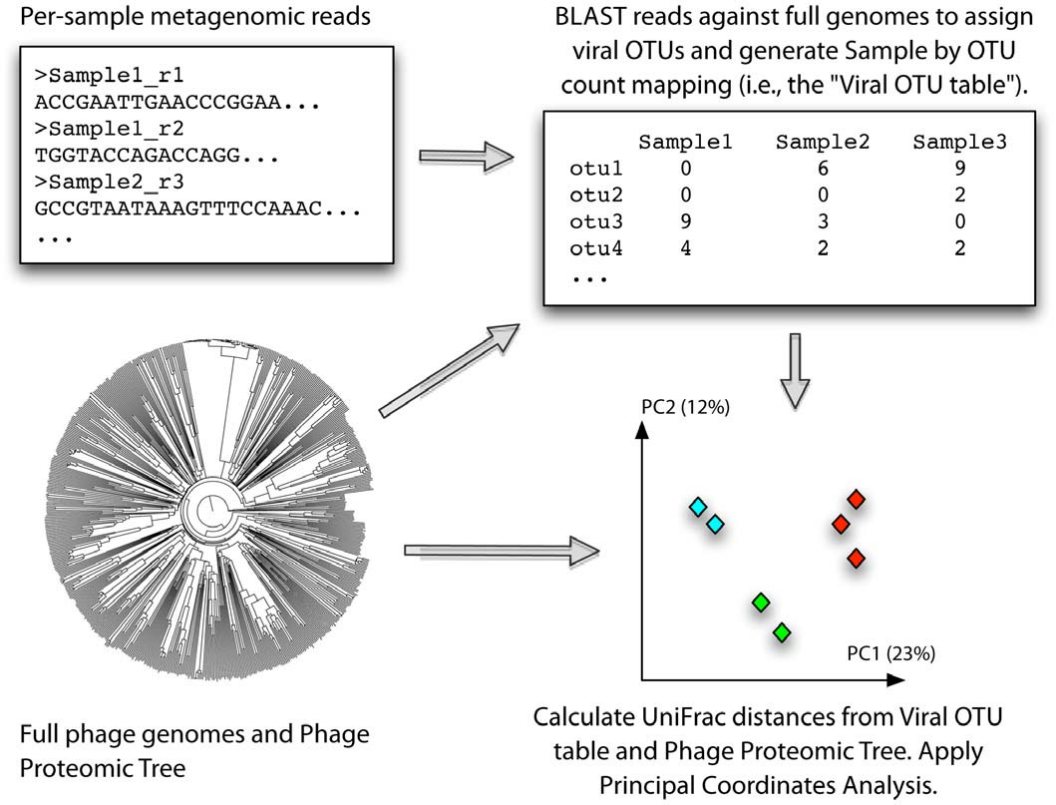
Schematic of the Shotgun UniFrac analysis pipeline.

The lack of a single marker gene in viral genomes complicates phylogenetic profiling of viral communities, a powerful technique for studying microbial communities, and previous studies have focused on profiling viral gene functions [4]. To complement these data with phylogenetic profiles of phage community composition, we developed Shotgun UniFrac (Figure 1). Shotgun UniFrac matches metagenomic reads against full phage genomes from the Phage Proteomic Tree [5] using BLAST. OTUs are assigned to reads by best hit, discarding reads with no significant hit, and UniFrac is applied using QIIME [6] and the Phage Proteomic Tree.

## Results

**Figure 2:**
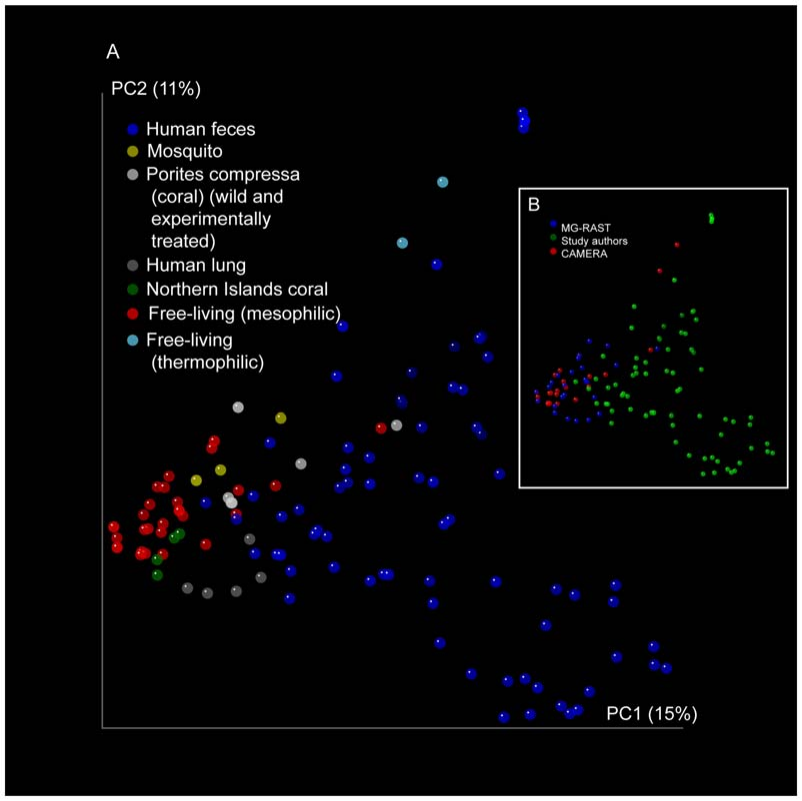
Principal Coordinates plot of weighted Shotgun UniFrac distances between viral communities where each point represents a metagenome colored by (a) host type and (b) data source.

We applied Shotgun UniFrac to 130 phage metagenomes from diverse environments. As observed with microbial communities, the primary factor separating metagenomes was whether they were derived from a free-living or host-associated environment. Host-associated environments vary more than a variety of free-living communities (considering only matches to the subset of viruses in the reference tree), and phage communities from the same host species tended to cluster (Figure 2a).

**Figure 3:**
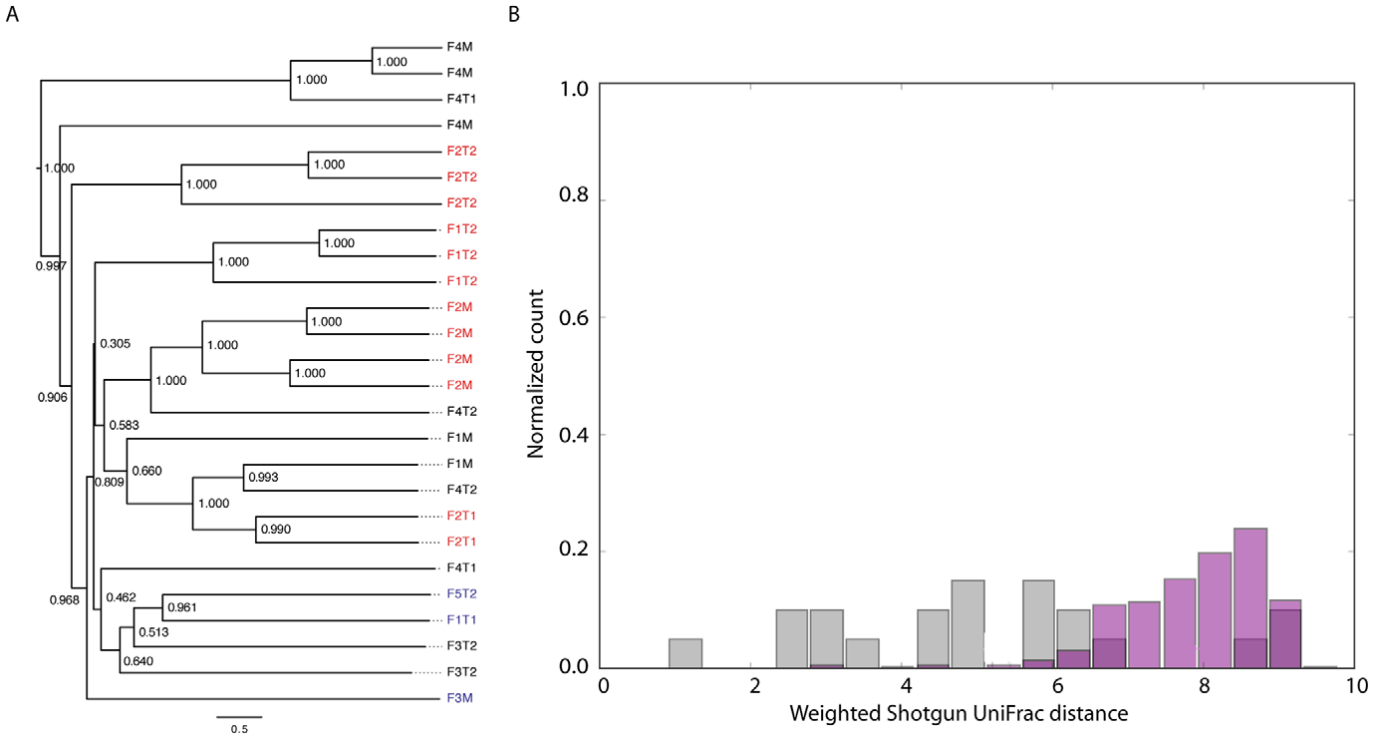
(a) UPGMA clustering of individuals by weighted Shotgun UniFrac distances between metagenomes. Cases where metagenomes from a single individual cluster monophyletically are highlighted in red. Cases where only a single metagenome for an individual was included are highlighted in blue. 1000 jackknife iterations were performed at a depth of 200 sequences per metagenome, and jackknife support values are provided for each node. The Reyes *et al.* analysis from which these samples were derived studied gut microbial communities from human twins and their mothers. The labels for each sample indicate the individual where: *Fn* corresponds to family number *n*; *M* corresponds to mother; and *T1* and *T2* refer to twin 1 and twin 2, respectively. (b) Histograms of within individual (grey) and between individual (pink) Shotgun UniFrac distances.

Our analysis also included 26 human feces phage metagenomes from 12 individuals with between 1 and 4 metagenomes per individual (recently presented in [7]). To include a metagenome in this analysis, we required a minimum of 200 reads assignable to a viral genome. We observed clustering of metagenomes by individual, although some aberrant clustering occurred (Figure 3a). This is likely due to the limited number of phage genomes currently available, which limits the resolution of Shotgun UniFrac (see Discussion). Confirming the observations of [7], [8] we found between-individual Shotgun UniFrac distances to be significantly greater than within-individual distances (Figure 3b; p = 3×10^−23^, one-tailed t-test; p<0.001, Monte Carlo t-test with 1000 iterations), suggesting stability in distal gut phage community membership over time.

## Discussion

Taken together, our results suggest that phage communities mirror microbial communities, and that comparison of phage communities by phylogenetic identity of viral types, even with relatively few sequenced phage genomes available to assign sequences, can be a powerful complement to functional profiles of the communities. Collecting viral metagenomes, microbial metagenomes, and 16S reads from the same samples and comparing these data with techniques such as Procrustes analysis [9] will provide insight into fundamental parameters of microbial ecosystems, such as whether control occurs in a top-down or bottom-up manner.

Currently the limiting factor in applying Shotgun UniFrac to phage data is the availability of phage genomes, because sequences not matching known genomes are excluded from the analysis. For some metagenome types less than 1% of the viral metagenomic sequences could be classified (Table 1, Table S1) resulting in relatively few sequences per metagenome for comparing communities. The UniFrac results presented in Figures 2 and 3 are based on exactly 200 sequences per metagenome. Data sets of this size are useful for comparing microbial communities [10] and phage communities (Figure 2), but increasing the database of sequenced phage genomes and their phylogenies will further enhance the resolution of these techniques. Better resolution will aid understanding the complex dynamics and large compositional shifts seen in the human infant microbiome and virome [11], [12] that might be due to predator-prey cycling leading to chaos. Understanding such disruptions might be key to developing an understanding of probiotics and a wide range of time-variable diseases, such as Crohn's disease.

**Table 1:** OTU assignment statistics by metagenome type.

Metagenome Type	n	Mean fraction failed OTU assignments	St. Dev. fraction failed OTU assignments	Median fraction failed OTU assignments	Min fraction failed OTU assignments	Max fraction failed OTU assignments	Sequences (OTU assignment input)	Sequences (OTU assignment output)
**Free-living (thermophilic)**	2	0.9675	0.0040	0.9675	0.9635	0.9715	30,624	939
**Northern Islands Coral**	4	0.9851	0.0038	0.9848	0.9813	0.9893	1,079,057	17,433
**Mosquito**	3	0.9898	0.0016	0.9909	0.9876	0.9910	1,612,878	16,814
**Human Feces**	81	0.9908	0.0104	0.9929	0.9418	1.0000	1,357,353	12,616
**Porites compressa (coral)**	6	0.9890	0.0068	0.9931	0.9760	0.9941	238,123	2,567
**Free-living (mesophilic)**	32	0.9931	0.0037	0.9934	0.9819	1.0000	7,471,890	52,432
**Human Lung**	5	0.9970	0.0001	0.9970	0.9970	0.9971	1,728,378	5,112

## Materials and Methods

Viral community metagenomic data was compiled from CAMERA [13], MG-RAST [14], and study authors [7] (Table S2, Table S3). There was no community clustering by data source (Figure 2b). Sequences were assigned to source viral genomes using Shotgun UniFrac, an extension of the reference-based OTU picking strategy presented by [15], using the open source QIIME and PyCogent [16] toolkits. Shotgun UniFrac was applied against full phage genomes from the Phage Proteomic Tree, and the associated reference tree was used for phylogenetic beta diversity analysis. Sequences were assigned to a viral genome if they achieved an E-value of less than 0.001, resulting in the viral OTU table (Table S4). The viral OTU table was then sub-sampled to 200 sequences per metagenome (Table S5) to control for depth of coverage. The UniFrac diversity metric was applied to the sub-sampled viral OTU table using the Phage Proteomic Tree. The version of the Phage Proteomic Tree used here contains 651 tips built from fully sequenced phage genomes as described in [5]. Community clustering and within- versus between-individual Shotgun UniFrac distances were calculated using Weighted UniFrac. Shotgun UniFrac analysis, Principal Coordinates Analysis, distance calculations and plotting were all performed using QIIME, and Shotgun UniFrac is accessible in QIIME v1.2.0-dev using the pick_reference_otus_through_otu_table.py workflow.

The number of input metagenomes by type were: Reclaimed water at discharge point (n = 1); Reclaimed water at point-of-use (n = 2); Freshwater stromatolite (n = 2); Hot Spring, Yellowstone National Park (n = 2); Potable water (n = 1); Saltern (medium salinity) (n = 5); Ocean (db:MG-RAST) (n = 4); Saltern (high salinity) (n = 3); Northern Islands Coral (n = 4); Marine stromatolite (n = 1); Ocean (db:CAMERA) (n = 4); Freshwater (n = 4); Human feces (n = 80); Saltern (low salinity) (n = 3); Healthy human lung (n = 2); Mosquito-associated (n = 3); Cystic fibrosis human lung (n = 3); *Porites compressa* (coral, wild and experimentally treated) (n = 6). Four overlapping metagenomes (Ocean (db:MG-RAST) and Ocean (db:CAMERA)), were used as controls to ensure that the source database did not affect the clustering results which is possible, for example, if one required preprocessing that the other did not.

## Supporting Information

Table S1 OTU assignment statistics by metagenome.
(XLS)

Table S2 Description of metagenome types and sources.
(XLS)

Table S3 Full QIIME metadata mapping file.
(XLS)

Table S4 Full viral OTU table (i.e., metagenome × viral OTU abundance matrix). These data were used in jackknifed weighted Shotgun UniFrac calculations (Figure 3a).
(XLS)

Table S5 Viral OTU table sub-sampled to 200 sequences per metagenome. These data were used in weighted UniFrac calculations (Figure 2 and Figure 3b).
(XLS)
